# Respiratory Trajectories and Correlation with Serum Biochemical Indices in Spinal and Bulbar Muscular Atrophy

**DOI:** 10.3390/brainsci14111057

**Published:** 2024-10-25

**Authors:** Federica Ginanneschi, Caterina Bigliazzi, Flora Anna Cimmino, Stefania Casali, Pietro Pelliccioni, Emanuele Emmanuello, Elena Bargagli, Nicola De Stefano

**Affiliations:** Department of Medical, Surgical and Neurological Sciences, University of Siena, 53100 Siena, Italy; c.bigliazzi@ao-siena.toscana.it (C.B.); pelliccioni_p@yahoo.com (P.P.); bargagli2@gmail.com (E.B.); destefano@unisi.it (N.D.S.)

**Keywords:** blood analysis, Kennedy’s disease, spirometry

## Abstract

Background/Objectives: The primary life-threatening complication in spinal–bulbar muscular atrophy (SBMA) is ventilatory failure. The present study analyzes the longitudinal patterns of respiratory function tests over a follow-up of 11 years. Methods: We collected data from 9 genetically confirmed SBMA patients. Spirometric measurements [maximum inspiratory pressure (MIP), maximum expiratory pressure (MEP), and forced vital capacity (FVC)], serum biochemical indices and SBMA functional rating scale (SBMAFRS) were collected every 6 months for 11 years. An average time curve was utilized to assess the changes in both pulmonary tests and serum biochemical indices of the patients. One-way repeated-measures ANOVA was applied to assess statistical differences. The Spearman’s rank correlation coefficient was utilized to evaluate the correlations between the respiratory function tests and serum biochemical and clinical indices. Results: A progressive decrease was observed in the respiratory function tests; the slope of the linear regression was significantly non-zero (*p* < 0.0001) for all three time curves. A major decrease was observed for MEP (52%) and MIP (42%), while this was minor for FVC (25%). SBMAFRS score correlated with FVC (r = 0.27), MIP (r = 0.53) and MEP (r = 0.51). MIP and MEP correlated with creatine phosphokinase (r = 0.3, r = 0.25, respectively) and MIP with creatinine levels (r = 0.31). Conclusions: This longitudinal study shows a progressive decline of spirometric data throughout life in SBMA patients. The decline appears to be related to clinical deterioration and muscle denervation. Spirometric measures relative to maximal strength of the respiratory muscles (MIP and MEP) may have a better predictive value for pulmonary and muscular decline than FVC.

## 1. Introduction

Spinal and bulbar muscular atrophy (SBMA), also known as Kennedy’s disease, is a rare X-linked, adult-onset, neurodegenerative disorder. It results from a Cytosine–Adenine–Guanine (CAG) repeat expansion in exon 1 of the androgen receptor gene [1,2]. The main pathological feature of SBMA is a slowly progressive lower motor neuron degeneration in the spinal cord and brainstem, leading to limb and bulbar muscle weakness, fasciculations, and atrophy. Additional features include myogenic lesions and a multisystem involvement mainly due to androgen insensitivity, contributing to other signs and symptoms such as gynecomastia, erectile dysfunction, reduced fertility, and metabolic changes (see Manzano et al. [3] for review).

Postural tremors, cramps, fatigue, muscle twitching, and slurred speech often represent the first clinical manifestations of SBMA. With disease progression, patients additionally develop dysarthria, dysphonia, hanging jaw, tongue wasting, chewing difficulty, and impaired mobility.

SBMA courses are usually prolonged, with an average 20-year interval between the onset of symptoms and death [4]. The main life-threatening condition in SBMA is ventilatory failure caused by weakness of the bulbar and respiratory musculature [5]. However, the natural history of the respiratory deterioration has not been well described. The importance of this topic lies in the fact that the monitoring of respiratory function is indispensable for the long-term care of SBMA patients, being critical not only in the respiratory but also for the nutritional management of the patients.

Indeed, in SBMA subjects, symptoms of dysphagia are quite early, with an average onset at the beginning of the fifth decade [4]. Weight loss, choking, and coughing during swallowing are some of the symptoms experienced. As in amyotrophic lateral sclerosis, a percutaneous endoscopic gastrostomy (PEG) should be inserted before the patient’s ventilatory capacity is compromised in order to avoid respiratory crises during surgery [6]. Longitudinal assessment of respiratory function is therefore crucial for managing SBMA patients.

Only one longitudinal study has previously characterized respiratory function in SBMA subjects, showing correlations between motor function and both forced vital capacity (FVC) and peak expiratory flow (PEF) [7]. The present study aims to study the trajectories of respiratory function tests such as maximum inspiratory pressure (MIP), maximum expiratory pressure (MEP), and FVC across eleven years of observation. In addition, we have explored the correlation between pulmonary function tests and clinical and biochemical serum indices. The interest in the biochemical serum indices arises from the fact that in SBMA patients there is a high prevalence of metabolic and liver disorders, mainly nonalcoholic fatty liver disease and insulin resistance [3]. In addition, the muscle histopathology of SBMA patients demonstrates both neurogenic and myopathic changes [8]. Myogenic findings are consistent with creatine phosphokinase (CPK) levels, which are higher in SBMA disease than expected for a neurogenic disorder [9]. Serum levels of CPK and liver enzymes are elevated even before symptom onset [10]. By analyzing the correlations between biochemical serum indices and respiratory function tests, useful information could be uncovered.

Improved insight into the characterization of the natural history of respiratory function in SBMA could direct therapeutic management of the disease, enhance the timing of supportive care, and be utilized as an outcome indicator for patient follow-up or future treatment efficacy evaluations.

## 2. Materials and Methods

### 2.1. Patients

This is a longitudinal, retrospective study carried out on medical records of patients with SBMA genetically established, admitted to the Center of the Motor Neuron Diseases of Siena University Hospital over 11 years. Exclusion criteria were competing disorders that could interfere with the pulmonary function test values, cognitive or physical disabilities that hinder the performance of respiratory, and other coexisting conditions that could impact the SBMA prognosis. The local Research Ethics Committee gave its approval to this study.

### 2.2. Clinical and Serum Biochemical Analysis

We collected demographic and genetic data, age at symptom onset, and comorbidities. For clinical analysis, we used a validated, disease-specific functional rating scale for SBMA (SBMAFRS) [11,12]. As the SBMAFRS scale has been validated since 2015, the score in the first years of disease was obtained for each patient by analyzing both the medical records and individual items of the Amyotrophic Lateral Sclerosis Functional Rating Scale. The latter scale was administered to patients before SBMAFRS validation. The following serum biochemical indices were analyzed: creatinine, CPK, myoglobin (MYO), glycated hemoglobin (HbA1c), triglycerides (TG), total cholesterol (CHOL), alanine and aspartate aminotransferase (ALT, AST, respectively), gamma-glutamyl transpeptidase (GGT), bilirubin, alkaline phosphatase (APh), and uric acid. We chose to analyze the serum indices found to be most frequently altered in SBMA according to the literature [13]. There was no patient who followed a particular diet or altered their lifestyle during the eleven years of the study.

### 2.3. Pulmonary Function Tests

All participants started with regular lung function tests, including FVC in upright seated positions and MIP and MEP in the upright position. Respiratory physiotherapists conducted the assessments in accordance with the guidelines of the American Thoracic Society/European Respiratory Society [14]. FVC is the maximal volume of air exhaled with a maximally forced effort from a maximal inspiration. MIP and MEP were estimated using a non-deformable face mask. To retrieve the MIP, it was necessary to exhale to residual volume and then inhale with the most effort possible for at least 3 s. The MEP was regained by a similar process but in the opposite direction: inhaling and then forced exhalation. The final MIP and MEP values were determined by the maximum value of three maneuvers.

All parameters were illustrated as a percentage of predicted normal values [15,16,17]. The respiratory physiotherapist collected age, height, and weight during every visit.

### 2.4. Statistical Analysis

Data were tested for their normality (Kolmogorov–Smirnov test with Lilliefors correction) before choosing a parametric or non-parametric statistical test. Descriptive statistics for continuous variables are illustrated as the mean ± SD. If the *p*-value was below 0.05, statistical significance was assumed. The decline in respiratory function tests and biochemical serum indices of patients with SBMA over 11 years was evaluated using a mean time curve. An average time curve was utilized to assess the decrease in both pulmonary tests and serum biochemical indices of the SBMA patients through 11 years. A one-way repeated measures ANOVA was applied to assess statistical differences.

The Spearman’s rank correlation coefficient was utilized to evaluate the strength of association between the respiratory function test and serum biochemical and clinical indices.

The relation between spirometric data and both clinical and serum biochemical results was then studied by comparing the serum biochemical indices obtained with MIP and MEP values below and above 50% of the predicted value (respectively) and with an FVC below or above 75% of the predicted value. We chose an FVC of 75% based on the results obtained in amyotrophic lateral sclerosis, in which an FVC of 75% is considered the threshold for the beginning of noninvasive positive-pressure ventilation (NIPPV), based on the fact that hypoventilation at night was shown in patients with an FVC of over 75% [18].

Mann–Whitney test and unpaired t-test were utilized to find differences between the values.

All tests were performed with GraphPad Prism version 7.04.

## 3. Results

**Figure 1:**
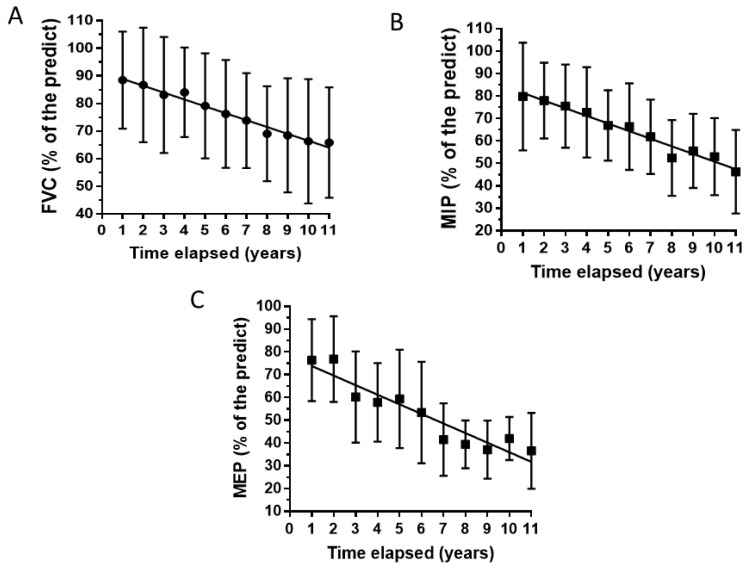
Average time curves for forced vital capacity (FVC) (**A**), maximum inspiratory pressure (MIP) (**B**), and maximum expiratory pressure (MEP) (**C**). The slope of the linear regression was significantly non-zero (F: 353, *p* < 0.0001 for FVC; F: 89, *p* < 0.0001 for MIP; F: 71, *p* < 0.0001 for MEP).

Nine subjects with SBMA were enrolled. The mean age at which the disease began was 47.78 ± 6.9 years, and the mean duration of the disease (calculated from the start of symptoms) was 17.8 ± 6.1 years. The CAG repeat number ranged from 42 to 50 (mean 45 ± 2.5). As expected [19], the CAG repeat number and age at disease onset were significantly inversely correlated (*p* = 0.0015; r = −0.91). At baseline, no patient complained of significant respiratory or swallowing deficits. All the patients had mild-moderate weakness, but none needed walking support. Comorbidities were bland steatosis in three patients, juvenile myoclonic epilepsy and Brugada syndrome in one patient, and epilepsy from childhood in one patient. Four out of nine patients never reported difficulty with breathing during the eleven years of observation; two reported dyspnea by the fifth year, two by the eighth year, and one by the seventh year. All five patients had mild dyspnea, which did not significantly affect daily life. Patients experience dyspnea during sustained exertion or occasionally during movements that involve bending the body, such as tying shoelaces. Two patients used NIPPV, both from the eighth year of observation. One patient died at the end of the eleventh year due to complications during PEG placement. Pulmonary function tests, blood chemistry tests, and the SBMAFS scale were performed/collected every six months for eleven years. Figure 1 shows the average time curves for FVC, MIP, and MEP. A progressive decrease was observed in all three respiratory function tests, with a statistically significant variation over the eleven years of measurements (one-way repeated measures ANOVA: *p*-values: 0.0036 for FVC and MEP and 0.0078 for MIP).

The average declines from the first to the eleventh year of observation were 25%, 42%, and 52% for FVC, MIP, and MEP, respectively. Table 1 reports the blood chemistry results.

**Figure 2:**
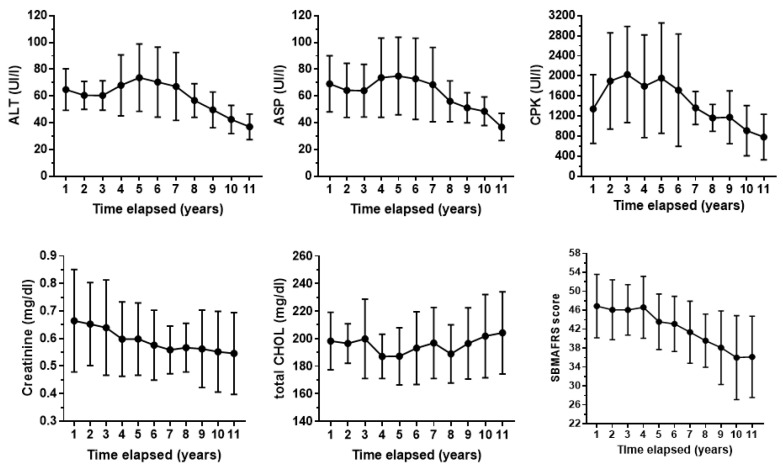
Average time curves for alanine and aspartate aminotransferase (ALT and AST, respectively), creatine phosphokinase (CPK), creatinine, total cholesterol (CHOL), and SBMAFRS (spinal bulbar muscular atrophy functional rating scale) score.

The values of CPK, MYO, ALT, and AST were altered in all patients, whereas GGT, bilirubin, and APh were normal, as well as the uric acid. HbA1 abnormality was a very rare event, and a minority of patients had a moderate increase in TG. Creatinine values were below the reference values in 55.5% of cases and the total CHOL in 66%. Figure 2 shows the average time curves for some serum biochemical parameters and SBMAFRS.

In the eleventh year of evaluation, the average decline with respect to the first assessment was 17% for creatinine, 43% for ALT, 44% for ASP, 41% for CPK, and 22% for SBMAFRS. The trend of creatinine is quite linear, while CPK and ALT/AST decrease after a period of increase.

**Figure 3:**
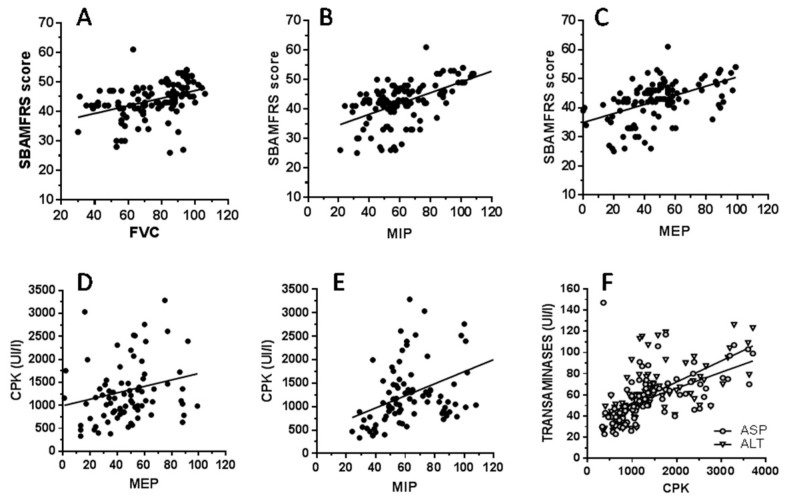
Correlation analyses of the pulmonary function test with clinical and serum biochemical values in 9 SBMA subjects. (**A**): r = 0.27; *p* = 0.0015; the slope of the linear regression was significantly non-zero: F = 23.13; *p* < 0001. (**B**): r = 0.53; *p* < 0.0001; the slope of the linear regression was significantly non-zero: F = 49.74; *p* < 0001. (**C**): r = 0.51; *p* < 0.0001; the slope of the linear regression was significantly non-zero: F = 42.72; *p* < 0001. (**D**): r = 0.25; *p* = 0.019; the slope of the linear regression was significantly non-zero: F = 4.51; *p* = 0.03. (**E**): r = 0.32; *p* = 0.0027; the slope of the linear regression was significantly non-zero: F = 16.2; *p* = 0.0001. (**F**): for ASP: r = 0.73; *p* < 0.0001; the slope of the linear regression was significantly non-zero: F = 36.33; *p* < 0.0001. (**F**): for ALT: for ASP: r = 0.74; *p* < 0.0001; the slope of the linear regression was significantly non-zero: F = 65.52; *p* < 0.0001.

The significant correlations between serum parameters, clinical score, and pulmonary function tests are reported in Figure 3.

There is a significant correlation between the SBMAFRS score and all three pulmonary function tests. MIP and MEP correlate with CPK. SBMAFRS score correlates with CPK (r = 0.24, *p* = 0.016). ALT and AST correlate only with CPK levels. Creatinine levels correlate with MIP (r = 0.31, *p* = 0.002) and SBMAFRS score (r = 0.23, *p* = 0.02); these data are not reported in Figure 3. The other serum indices have no correlations with anything.

We calculated the clinical and serum biochemical indices obtained with MIP and MEP values below and above 50% of the predicted value and with an FVC below or above 75% of the predicted value. SBMAFRS score was lower for FVC below 75% and for MIP and MEP below 50% (*p* = 0.0018, *p* < 0.0001, *p* < 0.0001, respectively). For MIP below 50%, the following serum indices were significantly lower: CPK (*p* < 0001), ASP and ALT (*p* = 0.03), and creatinine (*p* = 0.003). Only CPK and creatinine were significantly lower in the case of MEP below 50% (*p* = 0.01 for both).

## 4. Discussion

Spirometry is the most widely utilized volitional test for evaluating pulmonary volumes and capacity over time. Due to its importance in the diagnosis and management of disorders that affect the respiratory system, spirometry is included in the care recommendations for numerous neuromuscular diseases [20,21,22]. The lack of consideration for this issue in SBMA is likely due to the assumption that respiratory function is preserved in most patients with SBMA [5]. The present longitudinal study shows a progressive decrease in the values of MIP, MEP, and FVC over 11 years of observation in SBMA patients. Some of them (2 out of 9 patients) were severe enough to require the use of NIPPV. The SBMA group showed a close correlation between the three spirometric indices and the SBMAFRS score, suggesting that the clinical decline is closely linked to the worsening of spirometric data. The decrease in MEP and MIP values was severe, while it was mild for FVC. Indeed, measures of respiratory muscle strength decreased earlier than lung volumes in neuromuscular diseases [23,24], which is likely why they are more used than the measures of vital capacity in detecting respiratory muscle weakness [24,25,26].

MIP is closely related to diaphragmatic strength, while MEP is generated through the abdominal and intercostal muscles. In our study, we observed a correlation between spirometry indices and CPK levels for MIP and MEP but not for FVC. The lack of relationship was also confirmed by considering CPK values corresponding to FVC < 75%. As regards MIP and MEP, the relationship between CPK and these spirometric values is strengthened by the decrease in the latter. Among the other biochemical indices, the only other significant relationships were between ALT, ASP, creatinine, and MIP < 50%. All the other serum indices did not correlate with spirometric parameters.

Most CPK are located in skeletal muscle, and their elevation is affected by muscle mass and muscle damage; serum creatinine is a waste product from skeletal muscle creatine metabolism and is considered a predictive biomarker for SBMA [10,27]. Finally, the increased transaminases are likely derived from muscle because almost all of these patients had normal GGT, bilirubin, and APh. In this respect, the strong relationship between CPK and both transaminases and creatinine, as well as between CPK and MIP/MEP, lends support to the hypothesis that the spirometric parameters assessed here are related to muscle denervation.

Our results support the findings of the previous study on spirometry in SBMA patients, which demonstrated the superiority of PEF over FVC in highlighting spirometric alterations in SBMA [7]. PEF depends indeed upon the strength of the abdominal and intercostal muscles. On this basis, it is possible to believe that in SBMA, by using longitudinal data, spirometric measures relative to maximal strength of the respiratory muscles may predict pulmonary and muscle decline better than FVC. The temporal trend of serum values may be interesting since, despite the relatively low number of patients studied, these values were analyzed longitudinally, every 6 months for 11 years. Such extensive evaluations are present in the literature for a maximum of 3 years [28,29,30]. We showed that the serum levels of CPK, ALT, and AST are elevated in the early phase of the disease, while during the later stages of the disease, these levels gradually decrease. In the last few years of observation, transaminases in some patients even reached normal values. The SBMAFRS score correlates with spirometric tests as well as CPK, indicating that the decrease in the clinical score follows the decrease in CPK values. Our belief is that the decrease in CPK is caused by the progression of degeneration of the lower motor neurons, which in turn reduces muscle mass. The correlation between the decrease in spirometric indices and both CPK and transaminases indicates that the trend over time of these serum indices can provide indications of the evolution of the disease. It is worth mentioning that in SBMA, the increase in transaminases, especially of ALT, has often been attributed to nonalcoholic fatty liver disease [13], but in our cohort, it is not so.

Creatinine values have slightly declined through the years, while all the other biochemical indices did not show substantial changes, apart from a mild elevation of cholesterol. Our results are in line with those of Atsuta et al. [4] obtained in 263 SBMA patients in which age-related changes in laboratory data were analyzed. In accordance with the previous studies [28,30,31], we demonstrate that creatinine levels in SBMA are not correlated with those of CPK, as CPK values are a measure of muscle injury rather than muscle function.

## 5. Conclusions

We showed here that (1) respiratory failure in SBMA is not uncommon when analyzed through spirometry; (2) the decline of spirometric tests appears to be related to clinical deterioration and muscle loss; their longitudinal evaluation is crucial for managing patients with SBMA, particularly in regards to when to place PEG tubes; (3) MIP and MEP may detect respiratory insufficiency earlier than FVC, being likely related to muscle denervation; (4) CPK, transaminases, and spirometric data may be used as biomarkers for predicting response to treatments.

Being a monocentric study regarding a rare disease, the main limitation lies in the small patient sample in comparison to other larger case series. Consequently, we cannot completely rule out bias selection that may limit the generalizability of the findings. However, the long period of observation and analysis of patients can certainly be a strong point of this study.

## Figures and Tables

**Table 1:** Serum biochemical results of 9 SBMA subjects. For each subject, the measurements were performed every 6 months for 11 years.

	CPK (UI/L)	MYO (ng/mL)	ASP (UI/L)	ALT (UI/L)	GGT (UI/L)	APh (UI/L)	BIL (mg/dL)	CRE (mg/dL)	HbA1c (%)	CHOL (mg/dL)	TG (mg/dL)	UA (mg/dL)
Mean ± SD	1435 ± 877	387 ± 215	59.55 ± 23	62.68 ± 3	19.8 ± 7	51.04 ± 1	0.46 ± 0.2	0.6 ± 0.1	5.5 ± 0.4	200.6 ± 29	121 ± 45	5.2 ± 0.9
Range	331–4557	87–1152	23–124	26–128	8–42	22–101	0.13 ± 0.9	0.33 ± 0.9	4–6.2	145–295	43–227	2.8–6.8
Reference values	10–170	26–72	0–40	0–40	5–36	35–105	<0.15	0.50–1.1	4–6	<190	<150	3.4–7
no. of pt interested	9 (100%)	9 (100%)	9 (100%)	9 (100%)	1 (11%)	0	0	5 (55.5%)	1 (11%)	6 (66.7%)	3 (33.3%)	0

The percentages of abnormal values are calculated considering all the measurements. ALT, ASP: alanine and aspartate aminotransferase, respectively; APh: alkaline phosphatase; BIL: bilirubin; CHOL: total cholesterol; CPK: creatine phosphokinase; CRE: creatinine; GGT: gamma-glutamyl transpeptidase; HbA1c: glycated hemoglobin; MYO: myoglobin; no: number; pt: patient; TG: triglycerides, UA: uric acid.

## Data Availability

The data presented in this study are available on request from the corresponding author due to the fact that the statistical analyses and the data obtained were extrapolated from medical records and reports.
